# Inverse-designed ultra-compact high efficiency and low crosstalk optical interconnect based on waveguide crossing and wavelength demultiplexer

**DOI:** 10.1038/s41598-021-92038-w

**Published:** 2021-06-18

**Authors:** Yanhong Xu, Jie Huang, Lina Yang, Hansi Ma, Huan Yuan, Tong Xie, Junbo Yang, Zhenrong Zhang

**Affiliations:** 1 Guangxi Key Laboratory of Multimedia Communications and Network Technology, School of Computer, Electronics and Information, Guangxi University, Nanning, 530004 China; 2 Center of Material Science, National University of Defense Technology, Changsha, 410073 China

**Keywords:** Silicon photonics, Optoelectronic devices and components

## Abstract

In this paper, we use the inverse design method to design an optical interconnection system composed of wavelength demultiplexer and the same direction waveguide crossing on silicon-on-insulator (SOI) platform. A 2.4 μm × 3.6 μm wavelength demultiplexer with an input wavelength of 1.3–1.6 μm is designed. When the target wavelength of the device is 1.4 μm, the insertion loss of the output port is − 0.93 dB, and there is − 18.4 dB crosstalk, in TE_0_ mode. The insertion loss of the target wavelength of 1.6 μm in TE_0_ mode is − 0.88 dB, and the crosstalk is − 19.1 dB. Then, we designed a same direction waveguide crossing, the footprint is only 2.4 μm × 3.6 μm, the insertion loss of the wavelength 1.4 μm and 1.6 μm in TE_0_ mode is − 0.99 dB and − 1 dB, and the crosstalk is − 12.14 dB and − 14.34 dB, respectively. Finally, an optical interconnect structure composed of two devices is used, which can become the most basic component of the optical interconnect network. In TE_0_ mode, the insertion loss of the output wavelength of 1.4 μm at the output port is − 1.3 dB, and the crosstalk is − 29.36 dB. The insertion loss of the output wavelength of 1.6 μm is − 1.39 dB, and the crosstalk is − 38.99 dB.

## Introduction

With the advent of the information age, information storage capacity, transmission efficiency, and information processing capabilities have become critical elements^1^. The integrated optical system can greatly reduce the cost of device manufacturing and improve the information transmission capacity and has broad development prospects. The basis of on-chip optical interconnection^2–4^ is micro-nano photonic integration technology, of which the indispensable part is the wavelength demultiplexer and the waveguide crossing. Wavelength division multiplexing (WDM) is the core of on-chip optical interconnection. There are many wavelength demultiplexers are designed using traditional design methods. A wavelength demultiplexer based on SOI platform was designed^5^, with a footprint of 1.5 × 1.0 cm^2^ and crosstalk less than 25 dB. Based on the strong couplings of different Fabry–Perot (FP) resonators in metal–insulator-metal waveguides, a compact wavelength demultiplexer is numerically demonstrated with high wavelength resolution^6^. Furthermore, the authors of^7^ demonstrated a multichannel WDM designed, with a cavity diameter of approximately 300 nm or more and a final transmission rate of 70%. Although traditional design methods have lower insertion losses, they are often large in size, which is not conducive to densely integrated systems. Besides, the manual adjustment of parameters requires a lot of time and experience, it is difficult to achieve full-parameter space design^8,9^.

In recent years, the inverse design method of ultra-compact silicon photonic devices have drawn more and more attentions^10–12^. The three-channel wavelength demultiplexer^13^ has a pitch of 40 nm (1500, 1540, and 1580 nm), covering an area of 24.75 μm^2^. The final peak insertion loss is − 1.55 dB, and the crosstalk is less than − 15 dB. By using inverse design algorithms to explore the full-parameter space, we can realize nanophotonic devices with previously unattainable functionalities, or higher performance and smaller footprints than traditional devices^8,14,15^. In recent years, various wavelength demultiplexers^16,17^, and waveguide crossing structures^18^ have been designed using the inverse design method. In recent years, the algorithms most frequently used by researchers include direct binary search (DBS) algorithm^19,20^, genetic algorithm^21^, particle swarm optimization (PSO)^22^, and alternating direction method of multipliers (ADMM) algorithm^23^.

The idea of DBS algorithm is similar to the binary image optimization in computer data and image processing. Stanford University researchers have successfully designed a wavelength demultiplexer that can be realized in the 1300–1500 nm wavelength range^24^. A wavelength demultiplexer for CWDM system is designed^25^.The device experimentally displays low loss (− 2.3 dB), low crosstalk (− 16.4 dB), and broad 1-dB bandwidth (> 18 nm) with a compact size of 2.6 μm × 5 μm. Based on the previous research, we considered a wavelength demultiplexer with a wide communication bandwidth, ultra-compact, and low insertion loss. DBS algorithm is more convenient and fast than traditional design methods in the design of on-chip micro-nano optical devices. The device designed can not only greatly reduce the footprint of the device but also be easy to manufacture.

Similarly, the conversion of optical paths is bound to be involved. It is particularly important to reduce the insertion loss and crosstalk of the crossing of different optical paths, in the interconnection network. Therefore, waveguide crossing has attracted a lot of attention from researchers. ^26^ reported a multimode-interference waveguide crossing. Although the insertion loss is about 0.4 dB, its footprint of approximately 10.9 × 10.9 μm is not conducive to dense integration. The waveguide crossing is based on subwavelength gratings in silicon waveguides^27^. Its footprint is 3 μm × 10 μm, which is also very large. A dual-mode waveguide crossing^28^ supports the transmission of two modes at the same time. But the device requires two waveguides crossings and the footprint is very large, reaching 25 μm × 25 μm. These waveguide crossing designed using traditional methods also have the problem of large footprint. As a result, inverse design, with the DBS algorithm, has been used to design waveguide crossings. The authors in^29^ proposed a polarization-insensitive waveguide crossing by using an inverse design method. The ultra-compact dual-mode waveguide crossing based on the subwavelength multimode interference coupler is designed by using the DBS algorithm^30^ occupies an area of only 4.8 μm × 4.8 μm. The measured insertion loss and crosstalk are less than 0.6 dB and − 24 dB in TE_0_ and TE_1_ modes. ^31^ showed a waveguide bend and the waveguide crossing, they can all achieve low insertion loss, ultra-compact characteristics. However, they usually only discuss the input of the entire waveband and do not include waveguide crossing with different wavelengths that can be applied to the wavelength demultiplexer. In some proposed optical interconnection structures^32,33^, most of them discuss the routing of modes. Regarding the above design ideas, we use the DBS algorithm to design a basic optical interconnection system that includes a wavelength demultiplexer and a waveguide crossing with different wavelengths in the same direction. In the optimization process, the performance of the device is enhanced by adjusting the weight of the FOM and the discussion of the structural parameters. It can reduce the insertion loss and crosstalk after the device is integrated, and lays the foundation for the realization of any wavelength routing optical interconnection network.

In this paper, we first designed a wavelength demultiplexer with a footprint of only 2.4 μm × 3.6 μm, based on the SOI platform. Due to the reversibility of the optical path, a wavelength multiplexer can be obtained through asymmetrical structure designing. In the wavelength range of 1300–1600 nm, the insertion loss of the device at the output port of the target wavelength of 1.4 μm is − 0.93 dB, and the crosstalk is − 18.4 dB. At the same time, the insertion loss at the 1.6 μm output port is − 0.88 dB, and the crosstalk is − 19.1 dB. Besides, we also designed a waveguide crossing with different wavelengths in the same direction. The waveguide crossing of the designed structure occupies 2.4 μm × 3.6 μm, which is a very compact waveguide crossing. In the 150 nm working bandwidth centered at 1450 nm, the measured insertion loss and crosstalk of the corresponding wavelengths of the two ports are − 0.99dBand − 1 dB, and − 12.14 dB and − 14.34 dB, respectively. Then, an optical interconnection structure is designed using the proposed wavelength demultiplexer and waveguide crossing, which can be a basic optical cross-connect. Compared with the traditional optical interconnection, the integration density and gain of various on-chip optical systems have been greatly improved. Finally, on the 150 nm working bandwidth centered at 1450 nm, the output port insertion loss of the target wavelength of 1.4 μm is − 1.3 dB and the crosstalk is − 29.36 dB. The output port insertion loss of the target wavelength of 1.6 μm is − 1.39 dB and the crosstalk is − 38.99 dB.

## Results

### Optical interconnection component

#### Wavelength demultiplexer

**Figure 1:**
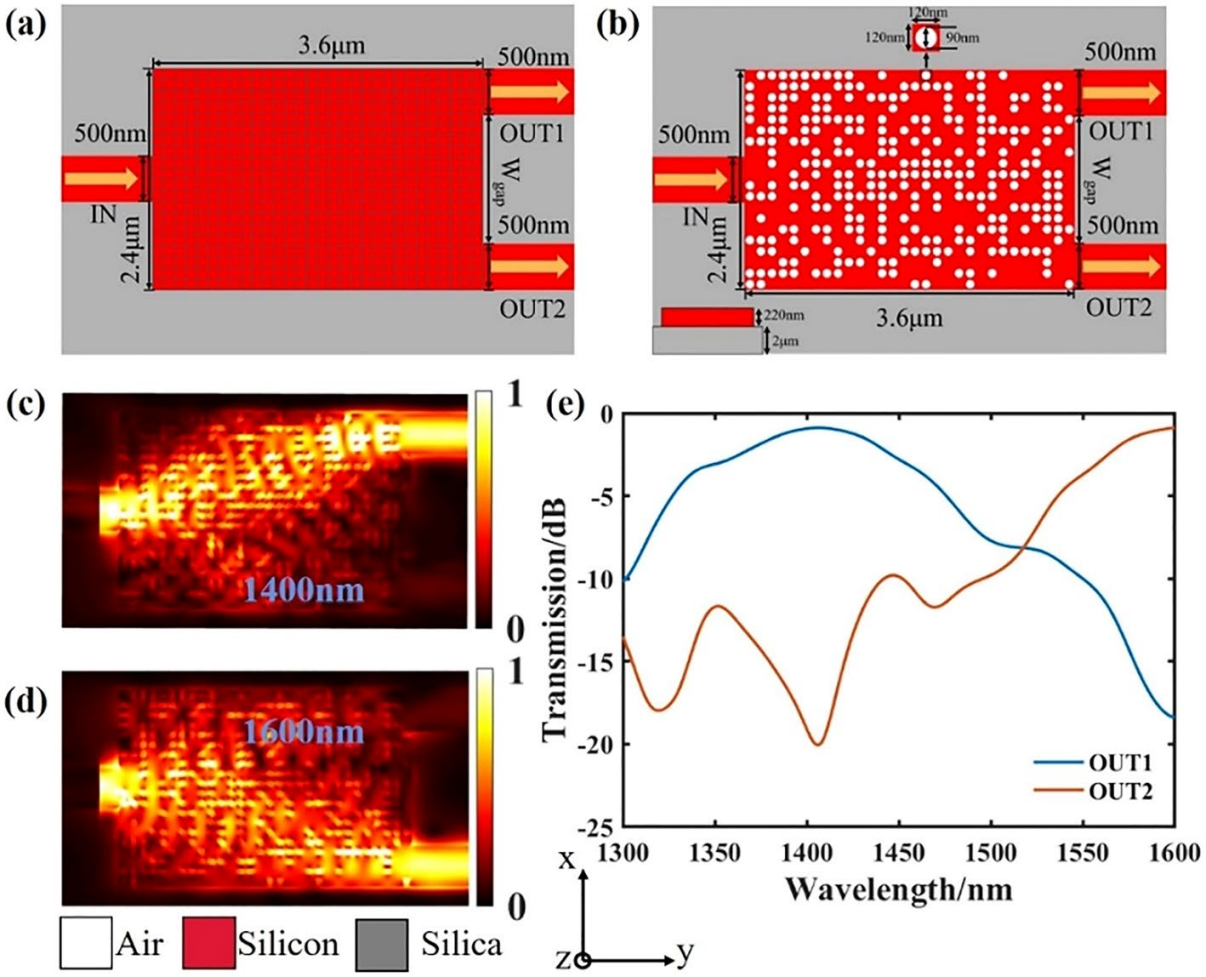
Design and simulation results of the wavelength demultiplexer. (**a**) Initial silicon slab before design optimization. (**b**) Final optimized structure. (**c**,**d**) are the simulated optical field distributions. (**e**) is the transmittance spectrum of each output port.

Based on the “method”, We first use DBS algorithm to design a wavelength demultiplexer on the standard commercial SOI substrate. The thickness of the silicon core layer is 220 nm, and the under-cladding silicon dioxide is 2 μm. As shown in Fig. 1a, the photonic-like crystal material structure^34,35^ is used for design, and its footprint is 3.6 μm × 2.4 μm. The advantage of the photonic-like crystal material structure is that regardless of the arrangement of the circular holes optimized by the final algorithm, the final circular holes are independent of each other and have the same characteristics. Such a structure can effectively avoid the inconsistent etching depth caused by the hysteresis effect^36^.The coupling area is composed of 20 × 30 pixels divided into a 120 nm × 120 nm square. Each pixel has two states. In the etched state, the radius of the corresponding central cylinder is 45 nm and the depth is 220 nm. The material is air, which is shown in white. When it is in complete state, the material is silicon, indicated in red. Then, to change the state of each pixel, we define an indicator parameter, figure-of-merit (FOM), to measure the performance of the target device. FOM is defined as:

1 $$ \begin{aligned} \text{FOM} & = a_{1} \left( {T_{{11}} + T_{{22}} } \right) + a_{2} \left( {1 - T_{{12}} + 1 - T_{{21}} } \right) \\  & \quad + a_{3} \left( {1 - \left| {T_{{11}} - T_{{22}} } \right| - \left| {T_{{12}} - T_{{21}} } \right|} \right) \\ \end{aligned} $$

Among them,*T*_*11*_ and*T*_*22*_ respectively represent the transmittance of the target wavelength at the corresponding output port. *T*_*12*_ and*T*_*21*_ represent the transmittance of the undesired output port. The items of FOM correspond to insertion loss, crosstalk, and two-channel imbalance of the wavelength demultiplexer, respectively. *α*_*1*_, *α*_*2*_ and *α*_*3*_ represent the weights corresponding to the three indicators. If the FOM is improved, the state of the pixel remains the state after the change. If the FOM is not improved, the state of the optimization unit returns to the state before the change. When all the pixels have gone through the above process, the algorithm is complete, which is an iteration. For each iteration, the algorithm stops until FOM converges.

We utilize FDTD solutions to solve the problem. In the wavelength range of 1300–1600 n, the input and output modes are both TE_0_ mode. For the wavelength demultiplexer, the final optimization result is obtained after 4 iterations, as shown in Fig. 1b. The target wavelength of output port 1 is 1.4 μm, and the target wavelength of output port 2 is 1.6 μm. The performance test of the final structure is performed, and the simulated optical field profile of the device shown in Fig. 1c,d is obtained. It can be seen that the target wavelength is well output from the target output port. Here, the insertion loss (IL) is defined as:

2 $$ \text{IL} = 10 \times \log \left( {\frac{t}{T}} \right) $$

Here *t* and *T* correspond to the transmittances of each output port and the input port the of the device. At the same time, we define crosstalk (CT) as:

3 $$ \text{CT} {\text{ = }}10 \times \log \left( {\frac{t}{T}} \right) $$

where *t* denotes the transmission efficiency of the target wavelength of the non-target output port, and *T* is the transmittance of the input port. Figure 1e shows the transmittance spectrum of the last two output ports. Finally, the insertion loss of output port 1 is − 0.93 dB, and the crosstalk is − 18.4 dB. The insertion loss of output port 2 is − 0.88 dB, and the crosstalk is − 19.1 dB. Due to the reversibility of the optical path, when light is input from the output end of the demultiplexer and output from the input end of the demultiplexer, and the pixel structure is horizontally symmetrical, the device can realize the multiplexing function.

**Figure 2:**
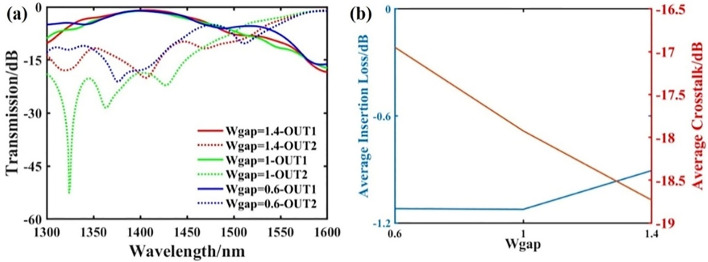
The effect of output waveguide spacing change on device performance. (**a**) The transmittance spectrum of the wavelength demultiplexer under different output waveguide spacing conditions. (**b**) The average insertion loss and crosstalk of the two target channels under different output waveguide spacing conditions.

In addition, to obtain better performance, we discussed the effect of the output waveguide interval *W*_*gap*_ on the device performance under the same initial conditions and iteration times. Figure 2a,b show the transmittance spectrum under different output waveguide spacing *W*_*gap*_ and the average insertion loss and crosstalk of the two target output ports. The results show that as the fluctuation interval increases, the impact on the insertion loss is relatively small. However, when the waveguide spacing is reduced, the output of the two target wavelengths is more likely to have an impact, resulting in higher crosstalk.

#### A same direction waveguide crossing

**Figure 3:**
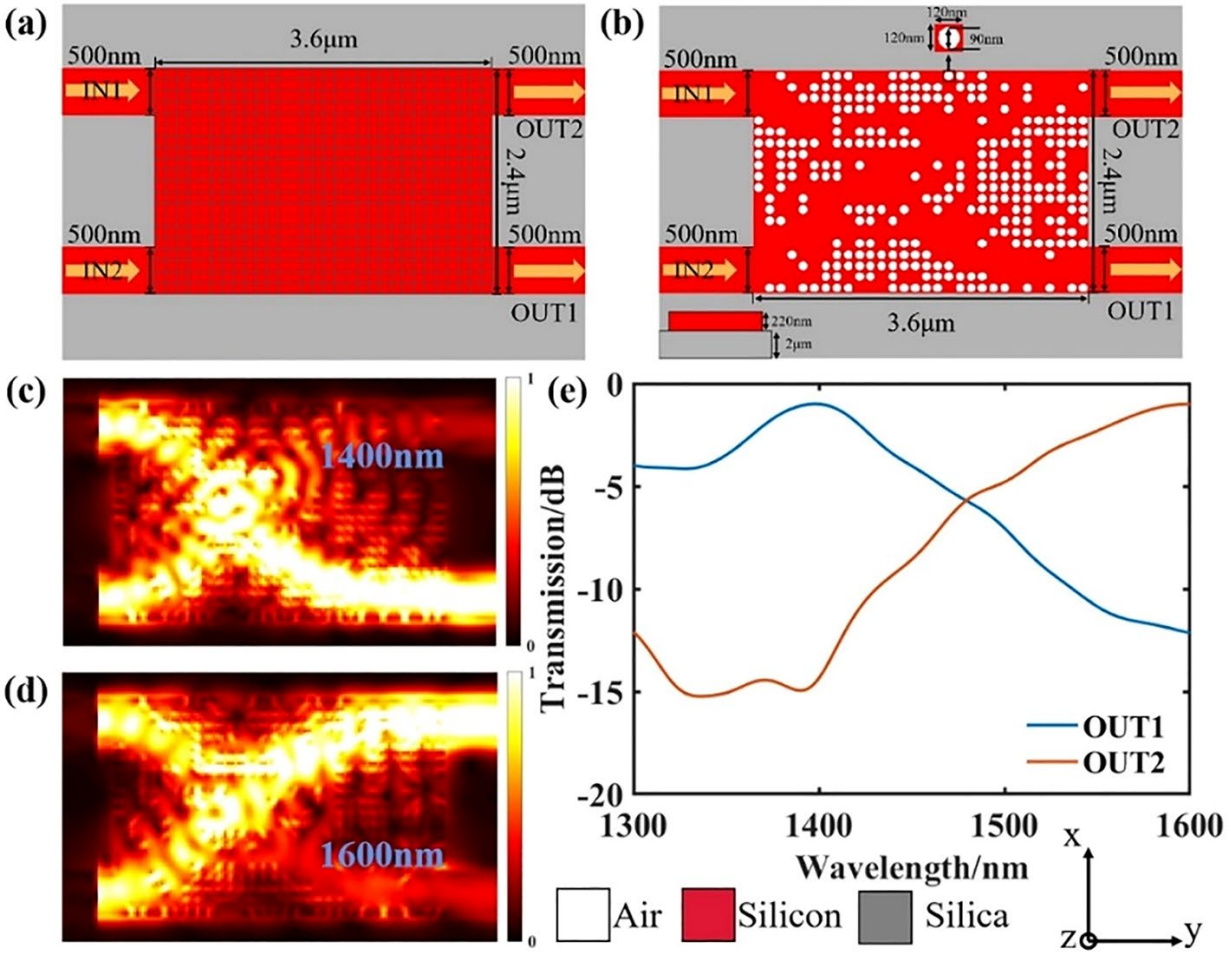
Design and simulation results of the same direction waveguide crossing. (**a**) Initial silicon slab before design optimization. (**b**) Final optimized structure. (**c**,**d**) are the simulated optical field distributions. (e) is the transmittance spectrum of each output port.

In the optical interconnection structure, it is indispensable to consider the crossing of optical paths to realize more optical path transmission possibilities. Here, we designed a waveguide crossing with different wavelengths in the same direction. This device can be connected with the wavelength demultiplexing to reduce the loss and crosstalk caused by wavelength cross in wavelength routing. The device also has a footprint of 3.6 μm × 2.4 μm. The initial structure diagram of the final device is shown in Fig. 3a. The width of the input and output waveguides is 500 nm, the distance between the two waveguides is 1.4 μm, and TE_0_ mode is supported. The waveguide cross uses the same optimization method as the above-mentioned wavelength demultiplexer. Here our FOM is also defined as Eq. (1), and the final structure is obtained after 4 iterations as shown in Fig. 3b. Figure 3c,d show the simulated light field distributions of 1.4 μm and 1.6 μm target wavelengths, respectively. Figure 3e shows the transmittance spectrum of output port 1 and output port 2, respectively. Finally, the insertion loss of output port 1 is − 0.99 dB, and the crosstalk is − 12.14 dB. The insertion loss of output port 2 is − 1 dB, and the crosstalk is − 14.34 dB.

**Figure 4:**
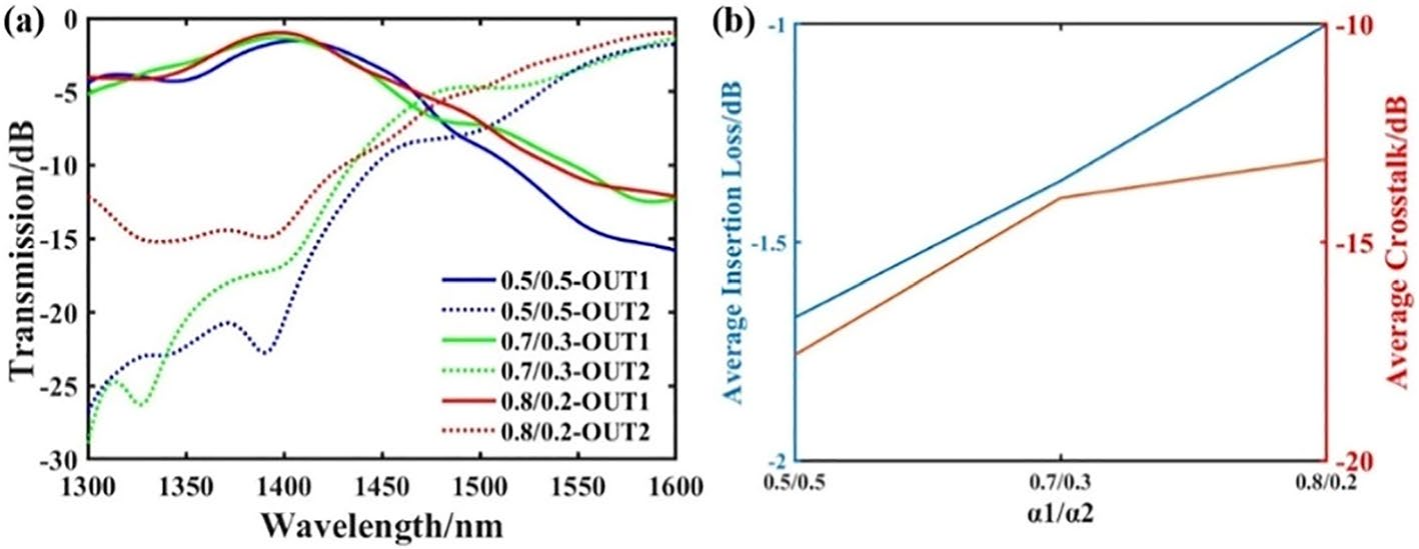
The effect of different weights changes on device performance. (**a**) The transmittance spectrum of the waveguide crossing under different weights. (**b**) The average insertion loss and crosstalk of the two target channels under different weights.

When we use the DBS algorithm, the final result depends on the weights *α*_*1*_, *α*_*2*_, and *α*_*3*_ in the FOM. Figure 4a,b show the changes in the insertion loss and crosstalk of the waveguide crossing at the two target wavelengths when *α*_*3*_ = 0.1 and different ratios *α*_*1*_/*α*_*2*_. We can analyze that when the weight *α*_*1*_ is relatively high, it means that we pay more attention to reduction in insertion loss and higher transmittance, when optimizing. At the same time, when the weight *α*_*2*_ is relatively high, it means that we pay more attention to the final crosstalk when we optimize, and the crosstalk will be lower. We can choose an appropriate ratio based on the actual situation. Finally, the ratio we choose for lower insertion loss is *α*_*1*_/*α*_*2*_ = 0.8/0.2.

### Optical interconnect structures

**Figure 5:**
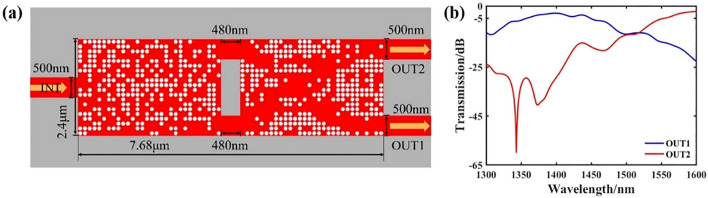
Design and simulation results of the initial structure after combination. (**a**) Schematic diagram of the structure. (**b**) is the transmittance spectrum of each output port.

**Figure 6:**
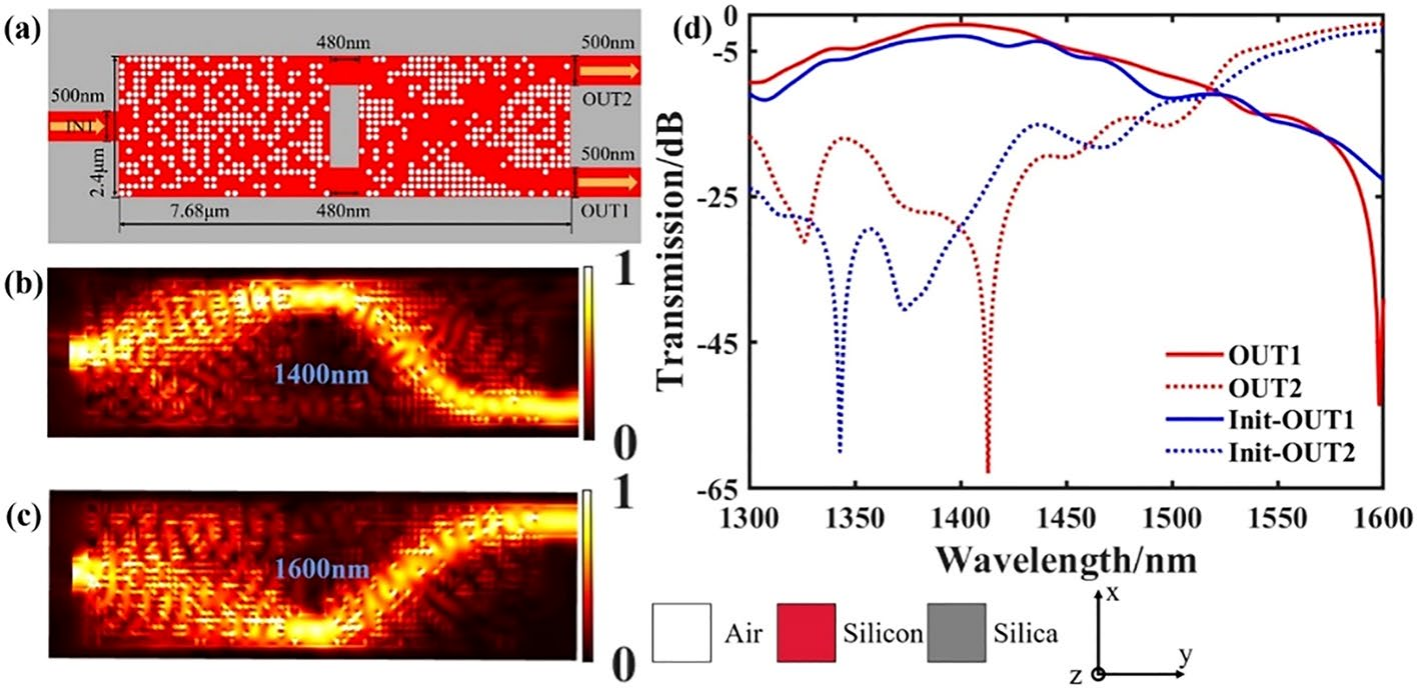
Design and simulation results of the optimized final structure. (**a**) The final structure after optimization. (**b**,**c**) are the simulated optical field distributions. (**d**) Comparison of transmission of each port of the optimized structure with the initial structure.

By combining the above-mentioned wavelength demultiplexer and a same direction waveguide crossing, a basic optical interconnection structure of wavelength cross-connection can be formed to realize the output of different paths of optical signals. The schematic diagram of the final structure is shown in Fig. 5a. The footprint of the device is 2.4 × 7.68 μm, where the distance between the two devices is 480 nm, and the width of the input and output waveguides are both 500 nm. The final transmittance spectra are shown in Fig. 5b. In the wavelength range of 1300–1600 nm, the insertion loss of the final output port 1 is − 2.22 dB and the crosstalk is− 29.25 dB. The insertion loss of output port 2 is − 2.96 dB and the crosstalk is − 22.71 dB. From the results, after we combined the two devices with a simple mechanical combination, the insertion loss was very high. Therefore, we take the structure of Fig. 5a as the initial structure and use the result of the wave decomposition multiplexer as the input port to optimize the pixel point structure of the waveguide crossing. The final structure diagram is shown in Fig. 6a. Figure 6b,c show the light field distribution of the target wavelength of 1.4 μm and 1.6 μm, respectively. The transmittance spectra are shown in Fig. 6d. In the wavelength range of 1300–1600 nm, the insertion loss of the final output port 1 is − 1.3 dB and the crosstalk is − 29.36 dB. The insertion loss of output port 2 is − 1.39 dB and the crosstalk is − 38.99 dB. It can be seen that through optimization, the final performance is improved.

**Figure 7:**
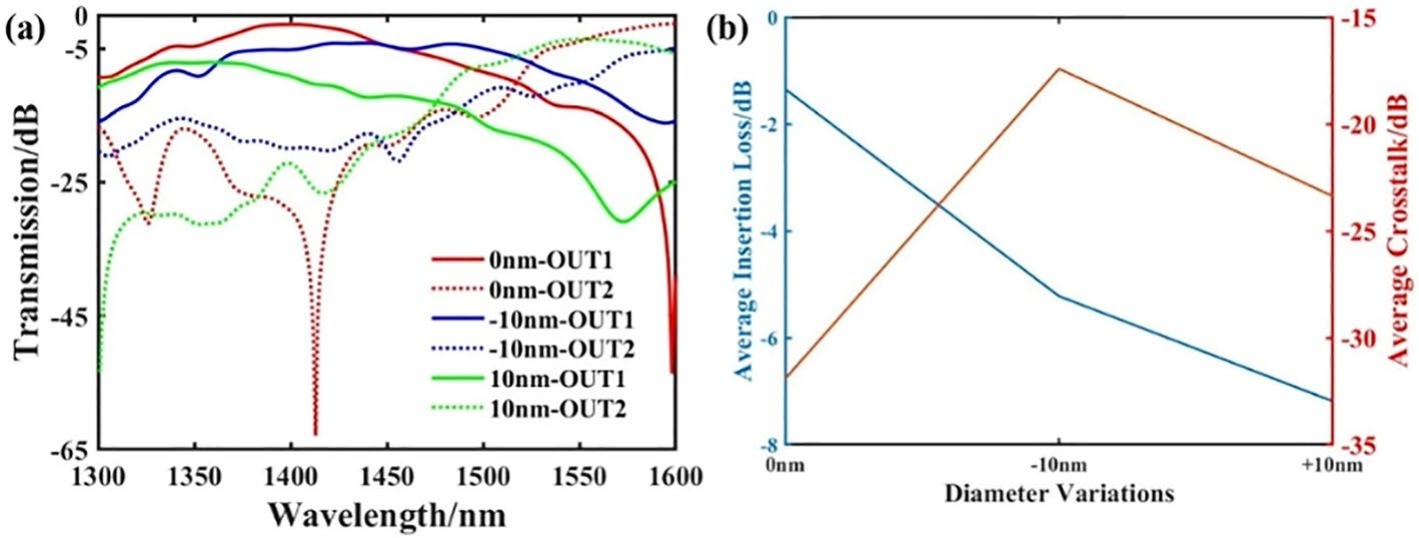
The transmittance spectrum under the diameter variations from − 10 to 10 nm. (**a**) The transmittance spectrum of output port 1 and port 2. (**b**) The average insertion loss and crosstalk of the two target channels under the diameter variations from − 10 to 10 nm.

Since some random and unavoidable manufacturing defects may appear in actual production, manufacturing tolerances must be considered. In Fig. 7a, we respectively show the transmission curves of output ports 1 and 2 when the diameter of the circular hole varies from − 10 to 10 nm. And we have plotted the average loss and crosstalk of the two target output ports in Fig. 7b.We can see that the change in the size of the hole has a significant impact on the insertion loss and crosstalk of the structure. We can conclude that larger apertures will affect the performance of these devices more, and we may get unexpected performance under large aperture changes from 10 to − 10 nm.

## Discussion

This paper uses the inverse design method and the DBS algorithm to design an ultra-compact, broadband optical interconnection based on waveguide crossing and wavelength demultiplexer on the SOI platform. The footprints of the waveguide crossing are only 2.4 × 3.6 μm. The insertion loss and crosstalk of port 1 of the wavelength demultiplexer are − 0.93 dB and − 18.4 dB, respectively, and the insertion loss and crosstalk of port 2 are − 0.88 dB and − 19.1 dB, respectively. The insertion loss and crosstalk of waveguide cross port 1 are − 0.99 dB and − 12.14 dB, respectively, and the insertion loss and crosstalk of port 2 are − 1 dB and − 14.34 dB, respectively. In the final optical interconnect structure, the insertion loss and crosstalk of port 1 are − 1.3 dB and − 29.36 dB, and the insertion loss and crosstalk of port 2 are − 1.39 dB and − 38.99 dB. The performance of the equipment is very good. In fact, by designing the basic optical interconnection structures, they can be used to implement more optical interconnection structures to better transmit optical signals. The optical interconnection structure we designed has high compactness, which is more conducive to the dense development of on-chip optical systems. At the same time, the design ideas and algorithms used in this article can be widely used in the research and design of optical devices.

## Methods

The overall process of inverse design consists of two parts. The first is the determination of device structure parameters and target performance. Secondly, according to the set performance requirements, various optimization algorithms are used to design and optimize the device. Here, we use DBS algorithm to simulate and compute the structure of our devices. Our specific design process is as follows. The device is first discretized into square pixels and each of size 120 × 120 nm. Considering the simplicity of the process, we chose the shape of the pixel block with a cylindrical structure (filled with air or silicon) at the center. The hole has a diameter of 90 nm. Next, defining figure-of-merit (FOM). Here, the FOM is defined as Eq. (1). The pixel state is retained if the FOM is improved. If not, the pixel state is reversed. Optimize continues until the FOM does not improve further.
